# Broader autism phenotype, the default mode network, and brain entropy: A network-level hypothesis

**DOI:** 10.1016/j.rin.2026.100044

**Published:** 2026-06-12

**Authors:** Souvik Dubey, Sujit Sarkhel, Samya Sengupta, Mahua Jana Dubey, Ritwik Ghosh, Julián Benito-León

**Affiliations:** a Department of Neurology, Bangur Institute of Neurosciences, IPGMER & SSKM Hospital, Kolkata, India; b Department of Psychiatry, Institute of Psychiatry, IPGMER & SSKM Hospital, Kolkata, India; c Consultant Psychiatrist, Berhampore Mental Hospital, Berhampore, India; d Department of General Medicine, Burdwan Medical College & Hospital, Burdwan, India; e Department of Neurology, 12 de Octubre University Hospital, Madrid, Spain; f Group of Neurodegenerative Diseases, Hospital Universitario 12 de Octubre Research Institute (i+12), Madrid, Spain; g Network Center for Biomedical Research in Neurodegenerative Diseases (CIBERNED), Madrid, Spain; h Department of Medicine, Complutense University of Madrid, Madrid, Spain

**Keywords:** Autism, Autism spectrum disorder, Broader autism phenotype, Default mode network, Functional connectivity, Triple-network model, Brain entropy, Social cognition, Sensory integration, Executive dysfunction, Endophenotype

## Abstract

Autism spectrum disorder (ASD) is highly heritable, and autism-related traits extend beyond diagnostic thresholds into the broader autism phenotype (BAP), a set of subclinical social-communication, cognitive, sensory, and personality-related characteristics observed in some relatives of autistic individuals and in the general population. The BAP literature is heterogeneous: many family and twin studies report elevated trait levels among first-degree relatives, whereas others report null, domain-specific, or subgroup-specific effects. We therefore frame BAP as a probabilistic marker of shared liability rather than a universal familial profile or a missed diagnosis. Here, we integrate behavioral, cognitive, genetic, and neuroimaging evidence and propose, rather than establish, a network-level model in which atypical default mode network (DMN) organization and altered coupling with salience and central executive networks may help explain links among sensory integration, executive rigidity, and socio-affective/mentalizing traits. We also operationalize brain entropy as a family of neuroimaging metrics that quantify the irregularity, variability, or complexity of neural signals (e.g., sample entropy, multiscale entropy, wavelet-based regularity, and Lempel-Ziv complexity). Entropy may provide a testable bridge between neural dynamics and BAP-related behavioral heterogeneity; however, direct evidence in BAP remains limited. We conclude by outlining testable predictions, limitations, and future directions for multimodal, developmentally informed studies.

## 1. Introduction and terminology

Autism spectrum disorder (ASD) is a heterogeneous neurodevelopmental diagnostic category characterized by differences in social communication and interaction, along with restricted and repetitive patterns of behavior, interests, or activities. Many autistic people also show sensory processing differences, language and communication differences, and attentional or behavioral dysregulation, with or without hyperactivity (Stanković et al., 2012; Lai et al., 2014). In this article, we retain “ASD” when referring specifically to diagnostic categories and diagnostic or genetic literature, and we use “autism,” “autistic people,” and “autism-related traits” where broader, neurodiversity-affirming language is appropriate. This choice reflects current discussions on language preferences while acknowledging that individual preferences vary (Kenny et al., 2016; Bottema-Beutel et al., 2021).

Historically, diagnostic subtypes included childhood autism, atypical autism, Asperger syndrome, and pervasive developmental disorder-not otherwise specified, which were later subsumed under the single ASD diagnosis. In 1943, Leo Kanner published the first systematic description of early infantile autism and noted parental characteristics that suggested familial liability (Kanner, 1943). Using robust individual-subject analyses, Jouravlev et al. reported that reduced language lateralization is associated with autism and, to a lesser extent, with autism-like traits in the general population (Jouravlev et al., 2020).

### Definition and phenotypic scope of the BAP

We define the broader autism phenotype (BAP) as subthreshold autism-related traits that may involve social-communication style, pragmatic language, cognitive rigidity, perfectionism, social reticence, sensory sensitivities, circumscribed interests, and related socio-affective differences. BAP is not a clinical diagnosis and should not be conflated with undiagnosed or “missed” autism. Rather, it is a research construct used to describe dimensional traits that occur more often, but not universally, among relatives of autistic individuals and also appear in the general population (Bolton et al., 1994; Landry & Chouinard, 2016; Morrison et al., 2018; Constantino & Todd, 2005; Hartley et al., 2019; Baron-Cohen et al., 2001; Hurley et al., 2007; Losh et al., 2009; Faso et al., 2016; Wainer et al., 2013; Esbensen et al., 2009; Anthony et al., 2013; Caruana et al., 2021).

Autism is phenotypically diverse, reflecting genetic heterogeneity as well as developmental and environmental influences. Difficulties in facial emotion recognition, Theory of Mind (ToM), and alexithymia are commonly reported in autism and may contribute to social communication challenges, although they are not universal. Building on Kanner’s early clinical observations, (Kanner, 1943) the BAP has attracted increasing attention as a pattern of subclinical traits consistent with shared genetic architecture (Bolton et al., 1994; Landry & Chouinard, 2016; Morrison et al., 2018).

Dawson et al. described multiple domains that help define the BAP from genetic, brain, and behavioral perspectives (Dawson et al., 2002). These include differences in face encoding/processing; reduced sensitivity to social affiliation or social reward, with consequent social motivation and reciprocity difficulties; motor imitation differences; memory alterations, particularly involving medial temporal lobe-prefrontal circuitry; planning and executive dysfunction; cognitive rigidity; and subtle language differences, particularly in phonological processing (Dawson et al., 2002; Lainhart et al., 2002; Wilson et al., 2010; Gerdts & Bernier, 2011; Holmboe et al., 2010; Bora et al., 2017; Tsai et al., 2017). Consistent with these domains, functional magnetic resonance imaging studies in parents with BAP traits have reported atypical activation of cortical and subcortical regions implicated in speech and language processing, including the left anterior insula, bilateral cerebellum and thalamus, left sensorimotor regions and supplementary motor area, and right superior temporal and supramarginal gyri (Billeci et al., 2016; Baddeley, 1992; Ackermann & Riecker, 2010; Ghosh et al., 2008; Hickok & Poeppel, 2007).

BAP traits are observed in the general population and across family studies (Constantino & Todd, 2005). Across several studies, at least one BAP trait has been reported in approximately one-fifth of relatives of children with ASD, with higher rates than controls across pragmatic language, personality style, social cognition, and executive-functioning domains (Piven et al., 1997; Landa et al., 1992; Piven et al., 1994; Eyuboglu et al., 2018; Folstein et al., 1999; Hughes et al., 1994, 1999a). Twin studies further support strong genetic contributions: concordance for ASD is higher in monozygotic than dizygotic twins, and concordance estimates increase when broader autistic traits/BAP are included (Ronald & Hoekstra, 2011; Constantino, 2014; Castelbaum et al., 2019; Colvert et al., 2015a; Frazier et al., 2014).

This evidence should be interpreted cautiously. The literature is heterogeneous, and several studies report no significant differences between relatives and controls, domain-specific effects, or subgroup-specific patterns rather than a uniform BAP profile (Bora et al., 2017; Robel et al., 2014; Scheeren & Stauder, 2008; Pisula et al., 2015). Sibling studies similarly include reports of lower scores than controls or no group differences on some tasks, indicating that BAP expression is shaped by age, sex/gender, ascertainment, measurement instruments, family structure (simplex versus multiplex families), and trait multidimensionality (Robel et al., 2014; Scheeren & Stauder, 2008; Pisula et al., 2015; Chopik et al., 2021). These inconsistencies do not negate the BAP construct; instead, they argue for a probabilistic, domain-specific model rather than a deterministic familial phenotype.

Beyond conventional BAP domains, Trevis et al. delineated four endophenotypes (socially unaware, aloof, pedantic, and obsessive) that were enriched in large multiplex ASD families (Trevis et al., 2020; Bernier et al., 2012; Wheelwright et al., 2010; De Groot & Van Strien, 2017; Hellriegel et al., 2017). BAP-related characteristics have also been reported in individuals with epilepsy and in families with ASD, supporting the possibility of shared mechanisms and pleiotropy (Richard et al., 2017; Piven & Palmer, 1999). NBEA has been implicated as a candidate gene in neurodevelopmental disorders with early generalized epilepsy phenotypes, raising the possibility of overlapping genetic liability relevant to both epilepsy and BAP (Mulhern et al., 2018; Bolton et al., 2011; Mouridsen et al., 2008).

Trait expression also appears to vary by developmental period and sociocultural context. Kurtz et al. reported that autistic traits were more strongly associated with anxiety and depression in Black college-aged adults (Kurtz et al., 2023), emphasizing that associations between BAP traits and mental health may be moderated by race, culture, stress exposure, and access to support. Age-related work further suggests that autistic-like traits can vary from young adulthood to older adulthood, although the mechanisms underlying these changes remain unclear (Chopik et al., 2021).

### Historical and family-context considerations

Historically, Bruno Bettelheim advanced the “refrigerator mother” hypothesis, suggesting that emotionally cold parenting contributed to autism. This theory is harmful, scientifically unsupported, and widely discredited; it is mentioned here solely to avoid repeating past explanatory errors and to underscore that parenting does not cause autism (Bettelheim, 1967). Contemporary studies of parental traits, parental mental health, expressed emotion, and parenting behaviors are best understood as research on family functioning, stress, and support needs, not as etiological evidence that parental behavior causes ASD (Baker et al., 2011; Weintraub, 2011; Ingersoll et al., 2011; Ingersoll, 2010; El-Bouhali-Abdellaoui et al., 2025).

The literature on “helicopter parenting” is not specific to BAP and should not be overinterpreted. It is relevant only insofar as overinvolvement, autonomy restriction, or heightened family stress can influence adjustment, anxiety, depression, decisional procrastination, coping, and adaptability in adolescents and emerging adults (Pistella et al., 2022; LeBlanc & Lyons, 2022; Turner et al., 2020; Wang et al., 2021; Wu et al., 2023; Gao et al., 2023; Vigdal & Brønnick, 2022). Within a BAP framework, parental rigidity, social reticence, anxiety, alexithymia, or pragmatic-language differences may affect family communication and the experience of caregiving, while child characteristics and parental stress may also influence parenting behaviors. These relationships are potentially bidirectional and should be studied without causal blame.

### Genetic liability, endophenotypes, and co-occurring traits

Twin and family studies demonstrate that ASD is highly heritable (Spence, 2004). Accordingly, genetically related family members of autistic individuals often show milder autism-related characteristics, commonly referred to as the BAP (Rubenstein & Chawla, 2018; Rubenstein et al., 2019). Importantly, when we discuss ASD liability across generations, we do not mean that the behavioral expression of parental BAP traits directly causes autism in offspring. Rather, BAP traits are associated with shared genetic liability across generations, including polygenic influences and, in some families, rare or damaging variants. Gene-environment correlations and family-level influences are plausible modifiers of developmental trajectories but remain insufficiently established as mechanisms of ASD causation (Nayar et al., 2021; Dong et al., 2023).

In a systematic review, Rubenstein and Chawla summarized BAP traits as including pragmatic language difficulties, reduced social reciprocity, cognitive rigidity, stereotyped behaviors, impaired socioemotional perception, and aloofness (Rubenstein & Chawla, 2018; Rubenstein et al., 2019). Ingersoll further showed that BAP is associated not only with verbal language differences but also with nonverbal communication difficulties; mothers with BAP traits reported more depressed mood and higher parenting stress than controls (Ingersoll et al., 2011; Ingersoll, 2010). Complementing these behavioral observations, Thomas et al.’s “over-pruning” hypothesis described a possible regression trajectory in autism beginning with sensory and motor changes, followed by increasing social disharmony (Thomas et al., 2016).

More recent work suggests that face perception and facial emotion-recognition abilities independently predict BAP traits (Gignac et al., 2024). Harwood et al. reported atypical audiovisual processing in adults with BAP features, as reflected in event-related potentials (Harwood et al., 2023). In college students, pragmatic language difficulties associated with BAP have also been linked to avoidant and anxious romantic attachment styles (Lamport & Turner, 2014). Using cluster-analytic approaches, Berthoz et al. found that parents of autistic individuals exhibited greater social anhedonia and alexithymia than controls, and that socio-affective impairments, which are common in ASD, were more prominent in parents with BAP traits (Berthoz et al., 2013; Leonardi et al., 2020; Szatmari et al., 2008; Wang et al., 2024; Rea et al., 2019).

Nayar et al. reported a closer correspondence between parent and child phenotypes for mothers than for fathers: higher ASD polygenic scores in mothers were associated with BAP traits and were more strongly related to ASD liability in their children (Nayar et al., 2021). These findings are consistent with a potential protective effect in women and reinforce the polygenic basis of ASD-related phenotypes within BAP (Nayar et al., 2021; Sucksmith et al., 2011; Piven et al., 1997; Kiruthika & Tiwari, 2022; King & Bearman, 2009). In 2021, McDonald proposed the concept of “broader autism phenotype constellations” (BAPCO) to incorporate sociocultural influences, with assortative mating, education, occupation, and co-occurring conditions acting as potential modifiers (McDonald, 2021).

Broader autism phenotype traits often co-occur with other conditions, including dyslexia, sensory hypersensitivity, and medical or psychiatric comorbidities, reflecting genetic heterogeneity, pleiotropy, and epigenetic modulation (Croen et al., 2015; Oxelgren & Myrelid, 2017; Feliciano et al., 2019; Taylor et al., 2021; Reis et al., 2017; Bailey et al., 1995; Hossain et al., 2020; Bolton et al., 1998; Fournier et al., 2010a). However, none of these co-occurring conditions shows complete concordance with autism. Flashner et al. reviewed epigenetic factors relevant to ASD and described links involving UBE3A, GABA receptor genes, and RELN, as well as dysregulated expression of epigenetic modifiers such as MeCP2 with downstream pleiotropic effects (Flashner et al., 2013). Dong et al. integrated polygenic risk, damaging de novo variants in ASD risk genes, and sex to model total ASD liability and its phenotypic spectrum, including BAP (Dong et al., 2023).

In a systematic review, Ruparelia et al. described behavioral, cognitive, and psychiatric endophenotypes in parents of autistic probands and highlighted the need for clearer operational definitions (Ruparelia et al., 2017). Overall, mild social/communication differences, rigid or aloof personality traits, and pragmatic language difficulties appear to be key socio-behavioral and cognitive endophenotypes with strong genetic associations; depression and anxiety are also frequently reported (Robel et al., 2014; Kalnak et al., 2012; Hanson et al., 2015; Colvert et al., 2015b; López-Mourelo et al., 2017; Grove et al., 2014; Cullen et al., 2008; Sarovic, 2021; Perfilyeva et al., 2022; Nenadić et al., 2024; Schmitt et al., 2019). Esler et al. described a subtle parent-child relationship in measures of insistence on sameness, suggesting heritability (Esler et al., 2018). Consistent with prior work, Kose et al. concluded that social skills and communication impairments in parents of autistic children are important indicators of BAP (Scheeren & Stauder, 2008; Kose et al., 2013; Mohammadi et al., 2012).

In a multivariate analysis, El-Bouhali-Abdellaoui et al. reported that fathers’ emotional symptoms were associated with their own BAP traits, whereas mothers’ emotional symptoms were more strongly related to their child’s emotional dysregulation (El-Bouhali-Abdellaoui et al., 2025). Recognizing BAP traits in parents may therefore help clinicians design more integrated, family-centered care (El-Bouhali-Abdellaoui et al., 2025). Wu et al. suggested that mothers of autistic children may retain relatively intact implicit mentalizing even in the context of poorer overall mental health (Wu et al., 2024). Novacek et al. further reported that reduced hedonic social pleasure (social anhedonia) predicts autistic traits more strongly than general anhedonia (Novacek et al., 2016).

Individuals with ASD and those with elevated autistic traits may show atypical sensory processing and autonomic responses, including differences in visual attention/eye-tracking measures and pupillary light reflexes; these indices have been associated with restricted and repetitive behaviors (Soker-Elimaliah et al., 2023; Elsabbagh et al., 2009a). As noninvasive markers, such indices may ultimately support earlier identification of BAP traits. In addition, Bolte and Poustka suggested that visual disembedding, a local processing bias, may represent a relatively specific component of the broader cognitive autism phenotype in parents (Bölte & Poustka, 2006). Whyte et al. reported that reduced gaze following is associated with BAP traits in a sex-specific manner, being more pronounced in males than in females (Whyte & Scherf, 2018; Elsabbagh et al., 2009b; Nayar et al., 2018).

Motor differences have also been described in autism and BAP, including delayed motor development, poor coordination, and fine motor difficulties (David et al., 2009; Ming et al., 2007; Fournier et al., 2010b). Ganai et al. reported that ASD-like traits in parents of autistic children were associated with reduced gait speed, which may represent a candidate motor endophenotype (Ganai et al., 2024). Parental BAP traits have also been linked to children’s emotion regulation (DeLucia et al., 2022).

### Neuroimaging evidence: DMN and triple-network organization

The central neural proposal of this review concerns the default mode network (DMN), but the current BAP-specific neuroimaging literature remains limited. Available evidence includes atypical activation of speech and language regions in parents with BAP traits, (Billeci et al., 2016; Baddeley, 1992; Ackermann & Riecker, 2010; Ghosh et al., 2008; Hickok & Poeppel, 2007) Electroencephalography-based connectivity differences in fathers of autistic children, (Mehdizadehfar et al., 2020) associations between functional connectivity and broader autistic traits in non-autistic children, (Beckerson et al., 2025) and links between superior temporal sulcus folding, network connectivity, and autistic-like traits in a non-clinical sample (Nenadić et al., 2024). These studies support the plausibility of network-level BAP markers, but they do not yet establish DMN dysconnectivity as a shared substrate of BAP.

The autism neuroimaging literature is broader and more consistent in implicating atypical DMN organization, although the direction of effects varies across development, sex, analytic method, task/rest condition, head-motion handling, and participant characteristics. DMN nodes such as the medial prefrontal cortex, posterior cingulate cortex/precuneus, temporoparietal junction, and lateral temporal cortex are relevant to self-referential processing, autobiographical memory, social cognition, and mentalizing (Padmanabhan et al., 2017). Studies report both hypo- and hyperconnectivity in ASD, supporting the view that “DMN dysregulation” should be treated as a family of developmental and context-dependent alterations rather than a single biomarker (Padmanabhan et al., 2017).

The triple-network model provides a useful organizing framework. In this model, the salience network (SN), default mode network (DMN), and central executive network (CEN) interact to support salience detection, internally oriented cognition, externally directed cognitive control, and flexible switching between mental states (Menon, 2011). Autism studies have reported altered connectivity within and between these networks, including salience-network findings that can classify and predict symptom severity in children with ASD and sex-related differences in SN-DMN-CEN connectivity among autistic youth (Uddin et al., 2013a, 2013b; Lawrence et al., 2020). We therefore hypothesize that BAP-related differences in social cognition, cognitive flexibility, and sensory integration may arise partly from altered interactions among these networks, but this remains to be tested directly in BAP cohorts.

**Fig. 1.**
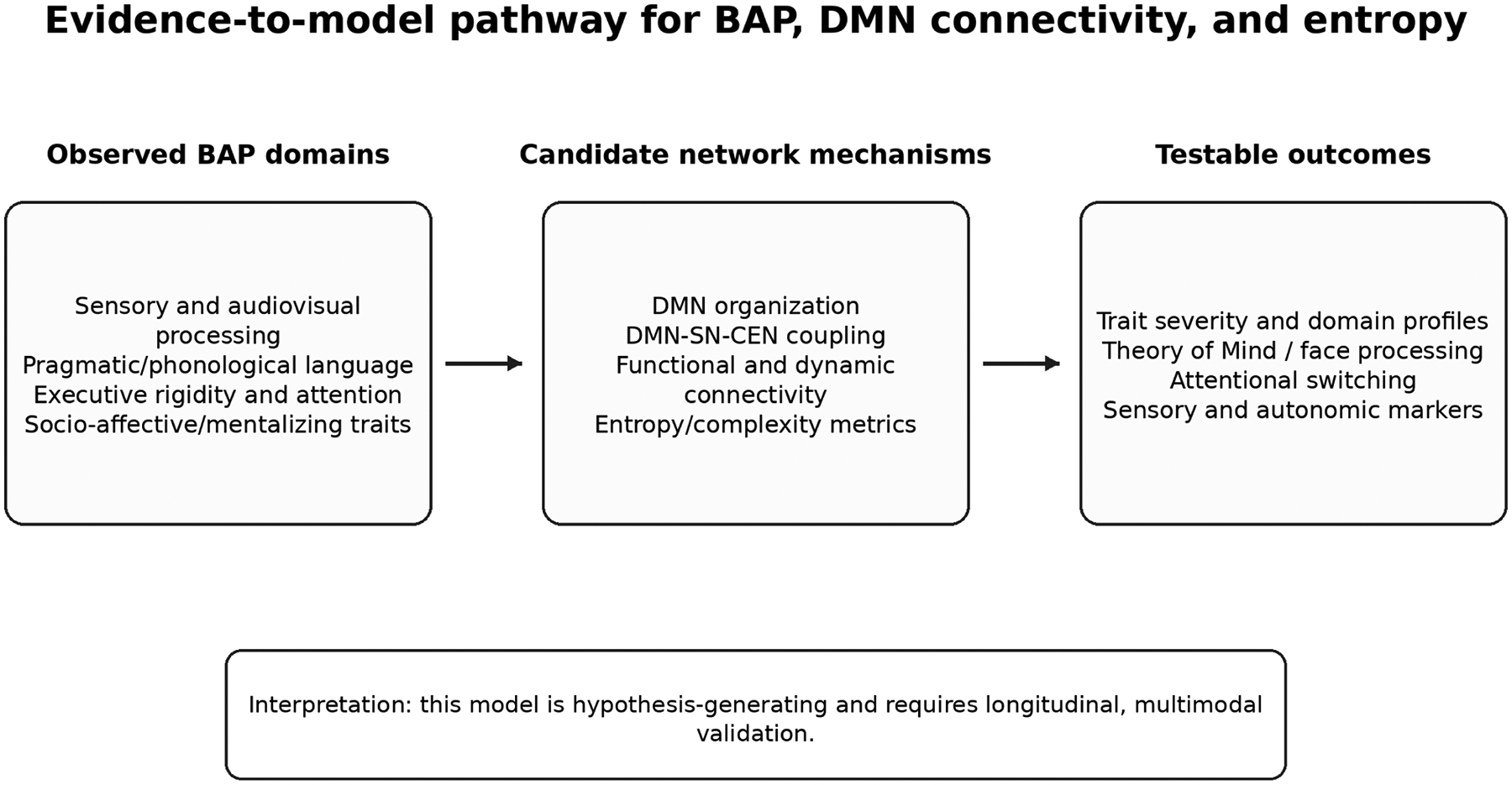
Evidence-to-model pathway linking observed BAP domains with candidate network mechanisms and testable outcomes. Abbreviations: BAP, broader autism phenotype; CEN, central executive network; DMN, default mode network; SN, salience network.

The evidence-to-model pathway linking observed BAP domains with candidate network mechanisms and testable outcomes is summarized in Fig. 1.

### Brain entropy: operational definition and relevance to BAP

In this review, “brain entropy” refers to quantitative neuroimaging metrics that describe the irregularity, variability, or complexity of neural signals over specified temporal and spatial scales. Entropy is therefore not used as a vague metaphor for randomness. In functional magnetic resonance imaging, electroencephalography, and functional near-infrared spectroscopy studies, commonly used approaches include approximate entropy, sample entropy, multiscale entropy, wavelet-based regularity/entropy, permutation entropy, and Lempel-Ziv complexity. These metrics differ from functional connectivity: entropy describes properties of a signal within a region or network, whereas connectivity estimates coupling among regions or networks.

Entropy findings in autism remain emerging and method-dependent. Smith et al. combined resting-state functional connectivity and wavelet-based entropy measures and reported that imbalanced connectivity across resting-state networks corresponded to a weakened relationship with temporal entropy; their entropy model also distinguished ASD from controls and was associated with symptom severity (Smith et al., 2018). Maximo et al. reported increased entropy in several cortical regions in ASD, including left angular gyrus, superior parietal lobule, and right inferior temporal gyrus (Maximo et al., 2021). These findings suggest that entropy may capture neural dynamics not fully explained by connectivity alone, but they do not establish a direct entropy-BAP or entropy-cognitive-resilience pathway.

Accordingly, our model treats entropy as a testable candidate mechanism. We hypothesize that atypical DMN dynamics and altered DMN-SN-CEN coupling may be associated with changes in signal complexity, which could, in turn, relate to variability in sensory integration, attentional switching, mentalizing, and executive rigidity. However, whether entropy reflects adaptive flexibility, inefficient neural noise, or compensatory dynamics may depend on developmental stage, brain region, metric, preprocessing, and behavioral context. Direct BAP studies are needed before entropy can be considered a BAP marker.

**Fig. 2.**
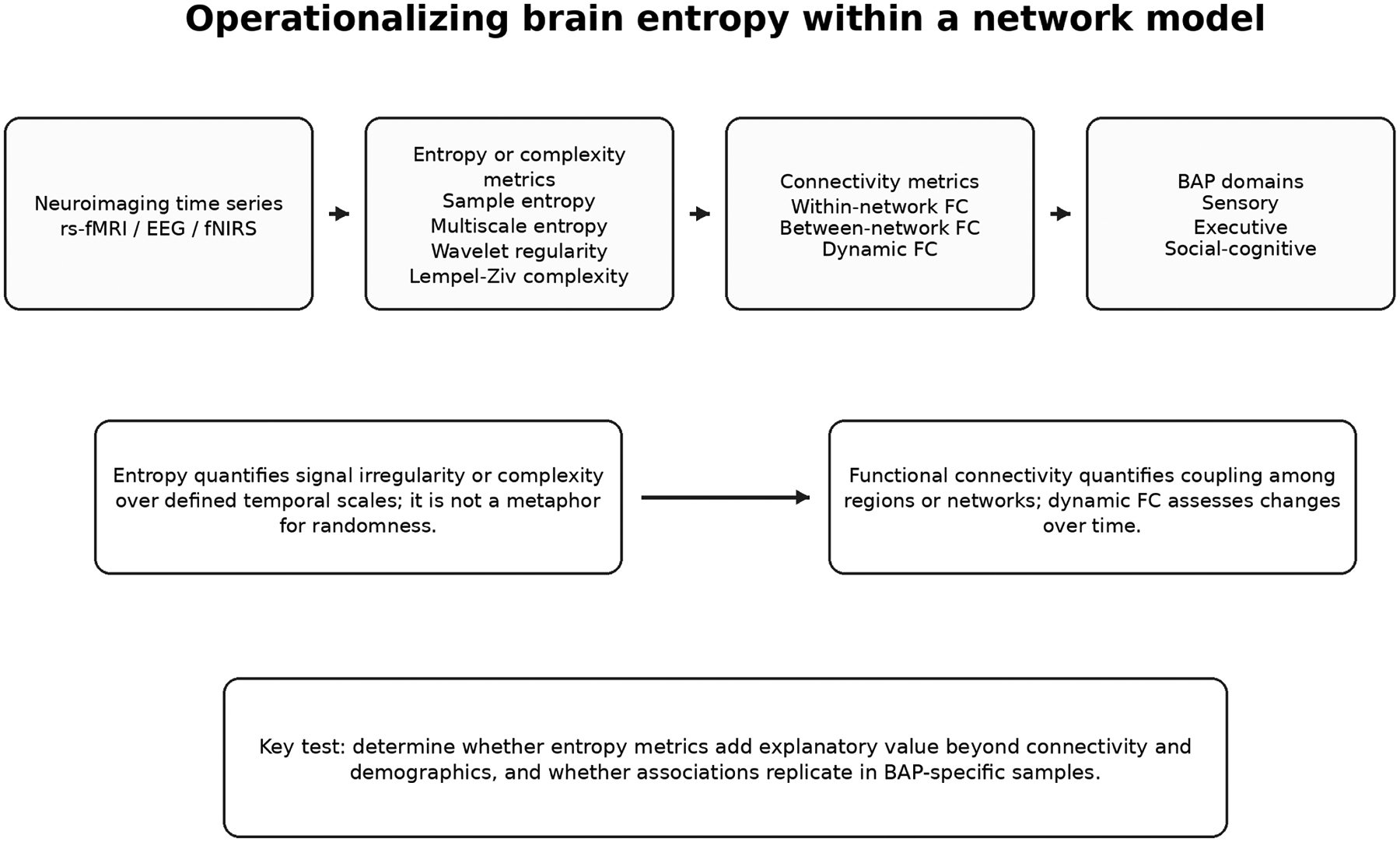
Operational framework for brain entropy in neuroimaging studies of autism-related traits. Entropy metrics quantify signal irregularity or complexity and should be interpreted alongside functional connectivity, dynamic connectivity, and behavioral measures.

An operational framework for incorporating entropy metrics into neuroimaging studies of autism-related traits is shown in Fig. 2.

### Authors’ hypothesis and testable predictions

Integrating the clinical characteristics and proposed endophenotypes of BAP, we propose a revised network-level hypothesis: BAP traits are associated with shared genetic liability across generations and may be expressed through heterogeneous behavioral, cognitive, and neural profiles. We do not claim that parental BAP traits behaviorally cause autism in offspring. Instead, we propose that BAP traits can index inherited liability and may interact with developmental and environmental factors that shape trait expression (Nayar et al., 2021; Dong et al., 2023).

**Fig. 3.**
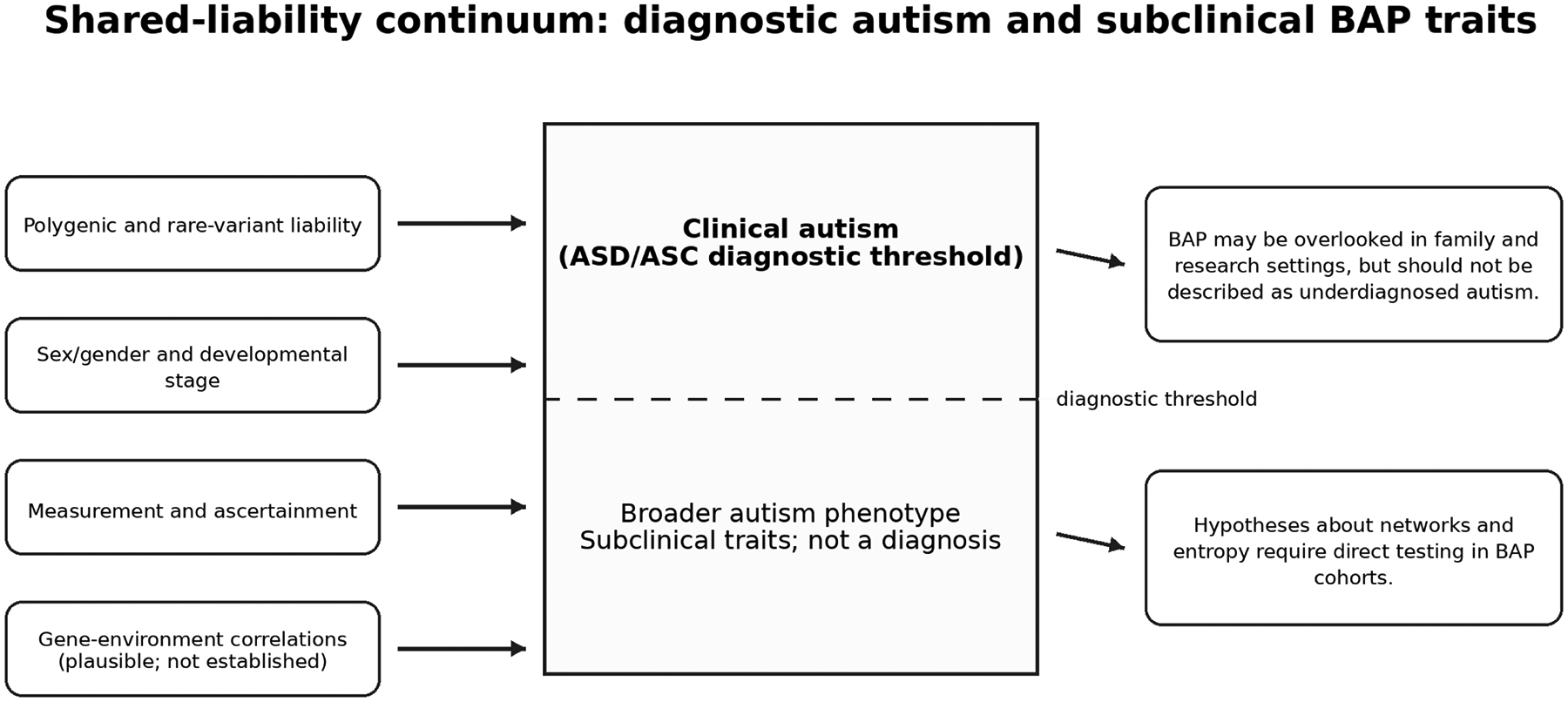
Revised shared-liability continuum model. BAP traits are subclinical and are not equivalent to underdiagnosed autism; they may nevertheless index shared liability and can be overlooked in family and research settings. Abbreviations: ASD, autism spectrum disorder; ASC, autism spectrum condition; BAP, broader autism phenotype.

The shared-liability continuum distinguishing diagnostic autism from subclinical BAP traits is summarized in Fig. 3.

Based on the available evidence, we propose a triad of interrelated BAP domains: (i) altered sensory processing and integration, potentially reflecting atypical coupling between unimodal and supramodal sensory cortices; (ii) subtle personality and behavioral styles characterized by social detachment, social unawareness, perfectionism, anxiety, or obsessive/pedantic traits, without implying categorical personality disorder; and (iii) altered functional organization of social-cognitive networks, contributing to differences in socioemotional perception, empathy, and ToM performance.

This model yields four testable predictions. First, BAP social-cognitive traits should correlate with DMN connectivity involving medial prefrontal, posterior cingulate/precuneus, temporoparietal, and lateral temporal regions. Second, rigidity, sensory intolerance, and attentional switching difficulties should relate to altered SN-CEN-DMN coupling. Third, entropy or complexity metrics should explain variance in BAP-related cognitive or sensory domains beyond that explained by static functional connectivity and demographic covariates. Fourth, associations should differ by age, sex/gender, family structure, and method of BAP ascertainment. These predictions are intended to guide research and should be evaluated in adequately powered, preregistered, longitudinal, and multimodal studies.

### Identification of BAP traits

Recent research has focused on identifying genetic and epigenetic modifiers in autism and BAP (Constantino & Todd, 2000; Loat et al., 2008; Lowe et al., 2015). Network-level connectivity alterations, particularly in the context of co-occurring neurodevelopmental conditions, have also been emphasized (Mehdizadehfar et al., 2020; Beckerson et al., 2025). In practice, however, BAP is typically recognized through convergent evidence across questionnaires, interviews, cognitive tasks, motor measures, language/pragmatic assessments, sensory measures, and family-history data. This multimodal approach is preferable because any single instrument may over- or under-detect traits depending on age, sex/gender, culture, and informant perspective.

Common markers include atypical sensory processing and integration; motor imitation differences; language and communication differences, especially phonological processing and pragmatic language; nonverbal communication differences; atypical audiovisual processing; less efficient face encoding/recognition; and social-cognitive differences such as reduced socioemotional perception, limited perspective-taking, and ToM differences (Glod et al., 2017; Bryant et al., 2019; Lewis et al., 2024; Tiede & Walton, 2021; Bojanek et al., 2025; Turbett et al., 2022; Sabatino DiCriscio & Troiani, 2017; Hughes et al., 1999b; West et al., 2020, 2018; Hickey et al., 2019; Schiltz et al., 2023; Arutiunian et al., 2024; Howlin et al., 2015; Stavropoulos et al., 2018; Gangi et al., 2021; Patel et al., 2023; Uljarević et al., 2021). Affective and behavioral features, including anxiety, depressive symptoms, obsessive or pedantic traits, alexithymia, cognitive inflexibility, and executive dysfunction, may co-occur. Subtle gaze abnormalities may also be present (Arutiunian et al., 2024; Howlin et al., 2015; Stavropoulos et al., 2018; Gangi et al., 2021; Patel et al., 2023; Uljarević et al., 2021).

### Limitations and future directions

Several limitations should temper interpretation. First, this is a theoretical narrative review, not a systematic review or meta-analysis. Second, BAP definitions and measures vary substantially across studies, and null findings or subgroup-specific effects are common. Third, direct neuroimaging evidence for BAP remains sparse compared with the broader ASD literature. Fourth, entropy metrics are sensitive to acquisition parameters, preprocessing, temporal scale, motion, and analytic choices; they should not be treated as diagnostic markers without replication. Fifth, neurodiversity-affirming research should avoid deficit-only framing and should include outcomes that are meaningful to autistic people and their families.

Future studies should combine genetic data, family-history methods, dimensional BAP measures, functional connectivity, dynamic connectivity, entropy metrics, and behavioral tasks in the same participants. Longitudinal designs are especially important for determining whether network and entropy measures predict trait stability, developmental change, compensatory mechanisms, or mental-health outcomes. Socio-cultural factors, including changing patterns of digital social engagement, may influence trait recognition or opportunities for social learning. Still, these ideas are speculative and should be tested empirically rather than assumed.

## 2. Conclusions

In summary, the strongest evidence supports BAP as a multidimensional and heritable set of subclinical autism-related traits rather than a uniform phenotype or an underdiagnosed form of autism. These traits span sensorimotor, cognitive, language/pragmatic, social-cognitive, and affective domains, and their expression varies across individuals, families, age, sex/gender, culture, and measurement approach. Neuroimaging studies in autism and preliminary work in BAP suggest that DMN organization and DMN-SN-CEN interactions are promising targets. Still, they are not yet established as shared substrates of BAP.

We therefore present DMN dysconnectivity and brain entropy as a hypothesis-generating framework. Brain entropy should be operationalized with established metrics and interpreted alongside functional connectivity, developmental stage, and behavioral context. Multimodal, longitudinal, and neurodiversity-informed research will be required to determine whether connectivity- and entropy-based measures improve characterization of BAP traits, clarify mechanisms of shared genetic liability, and guide family-centered supports without conflating BAP with clinical diagnosis or assigning causal blame to parents.
